# Living conditions of migrants in bavarian forensic psychiatry – acculturation, language competence and perceived ward climate

**DOI:** 10.1192/j.eurpsy.2021.1006

**Published:** 2021-08-13

**Authors:** L. Titze, M. Dudeck

**Affiliations:** Department Of Forensic Psychiatry And Psychotherapy Günzburg, Ulm University, Günzburg, Germany

**Keywords:** Migration background, acculturation, forensic psychiatry, language competence

## Abstract

**Introduction:**

From 2014 on, the rate of persons with a migration background in Germany rose steadily, in forensic psychiatric hospitals even disproportionally. Although daily work is aggravated through language barriers and cultural differences, general therapy processes have not been appropriately adapted yet and extensive research is lacking.

**Objectives:**

Therefore the aim was to get a picture of the current situation of patients with a migration background in 11 Bavarian forensic psychiatry.

**Methods:**

237 Patients with a migration background (first or second generation) were asked about their sociocultural background, their acculturation tendency (by rating the Frankfurter Acculturation Scale), their German language ability and their estimation of the ward climate (by the Essen Climate Evaluation Schema).

**Results:**

51.8% of the participants were able speak German on an A- level, 13.1% on a B- level and 35.1% on a C- level. Patients of our sample oriented themselves more towards Germany and less towards their country of origin, compared to the control sample. Further, they experienced safety significantly lower and patient cohesion and mutual support higher than the forensic reference sample.

**Conclusions:**

One possible explanation for the patients’ orientation is the lack of possibilities to act out their cultural traditions. Because of the patients’ limited German knowledge and cultural misunderstandings, they could feel less safe. To conclude, the group of patients with a migration background is important not solely due to its size. But its heterogeneity makes universally applicable statements not easy. Migration backgrounds have to be considered within the psychiatric setting and individual therapy plans have to be made.

